# Beyond the Outburst: Charting a New Frontier for Understanding and Treating Irritability in Autistic Adults

**DOI:** 10.1002/aur.70287

**Published:** 2026-06-01

**Authors:** Hsiang‐Yuan Lin, Arthur N. Westover, Amy S. F. Lutz, Manish K. Jha

**Affiliations:** ^1^ Azrieli Adult Neurodevelopmental Centre, Campbell Family Mental Health Research Institute, Centre for Addiction and Mental Health Toronto Ontario Canada; ^2^ Department of Psychiatry Temerty Faculty of Medicine, University of Toronto Toronto Ontario Canada; ^3^ Department of Psychiatry UT Southwestern Medical Center Dallas Texas USA; ^4^ Department of History and Sociology of Science University of Pennsylvania Philadelphia Pennsylvania USA; ^5^ O'Donnell Brain Institute, UT Southwestern Medical Center Dallas Texas USA

**Keywords:** autism, autistic adults, challenging behaviors, clinical trials, emotional dysregulation, irritability

## Abstract

Irritability is a prevalent and impairing feature associated with autism, yet remains poorly understood, particularly in adults. Drawing heavily on insights translated from pediatric and transdiagnostic literatures, we propose that irritability in autistic individuals often reflects a psychophysiological stress or threat response, rooted in a vulnerable neurobiology (e.g., sensory sensitivities, intolerance of uncertainty), but may also stem from intrinsic neurobiological dysregulation independent of environmental triggers. The current treatment paradigm for autistic adults, largely extrapolated from pediatric antipsychotic trials, leaves these adults critically underserved due to a lack of evidence‐based treatments, clinical trials, or validated tools to measure their internal experience. This viewpoint deconstructs irritability, differentiating its affective nature from aggression, and highlights heterogeneity across the lifespan and support needs. We critique the limitations of current assessment methods and recommend a shift toward a multi‐modal strategy integrating self‐report (when feasible) with objective, physiologically‐informed tools (e.g., wearable biosensors) and nuanced observer reports. We argue that the field is poised for neuroscience‐informed treatment innovation—including novel pharmacological agents and adapted psychosocial interventions—but is hampered by a lack of rigorous clinical trials in adults. Finally, we call for mechanistically driven trials to address the large unmet burden of inadequately treated irritability, ultimately improving the quality of life for autistic adults and their families/caregivers.

## Lay Summary

Irritability in autistic adults is a major crisis, but most treatments are decades old and were only tested on children. Drawing on evidence primarily from youth and related conditions, our review explains that irritability is often a physical and emotional stress response to things like sensory overload or trauma, not just a “behavior problem.” We call for urgent research into new, adult‐focused treatments and tools, like wearable sensors, to directly address the root causes of this distress.

## 1 Introduction

“What are we really treating when we prescribe risperidone or aripiprazole for ‘irritability’ in autism?” Two decades of drug approvals have not resolved this fundamental question. In clinical practice, the term “irritability” often serves as an imprecise shorthand, lumping together diverse phenomena (from fight‐or‐flight surges, anxiety‐fuelled or frustration‐induced meltdowns, to chronic hyper‐vigilance, some self‐injurious behavior (SIB), and aggression toward others) under a single symptom construct (Fung et al. 2016). Yet, each of these may reflect a distinct pathophysiology. We argue that the failure to disentangle these pathways, especially the circuit linking chronic stress and adverse experiences to physiological hyperarousal, has stalled progress for autistic adults, the very group most systemically excluded from the pivotal pediatric trials that define our current treatment paradigm.

The consequences of this conceptual and evidentiary gap are profound. Autistic adults navigate a clinical landscape that has limited understanding of their distress (Vohra et al. 2014) and a critical shortage of trained adult psychiatrists willing and able to provide care after the transition from pediatric services (Westover 2024). This “services cliff” often leads to crises, from repeated emergency visits (and/or hospitalizations) to the loss of community placements, with staggering economic costs (Cidav et al. 2012; Marsack‐Topolewski and Church 2019). Remarkably, despite irritability being the only FDA‐approved indication for pharmacological treatment in autism, no methodologically rigorous study has established the population‐level prevalence of clinically significant irritability specifically in autistic adults. Available estimates in the broader autism literature range widely from 19% to 80%, derived predominantly from pediatric or mixed‐age samples using heterogeneous methods (Lecavalier 2006; Lecavalier et al. 2019; Mayes et al. 2011; Mayes et al. 2019). The most rigorous adult‐population study of irritability to date used the Brief Irritability Test (BITe) (Holtzman et al. 2015) in over 42,000 US adults but did not characterize autism status (Perlis et al. 2024). The absence of an adult prevalence anchor for the very symptom that drives most pharmacological treatment in autism is itself emblematic of the field's systemic neglect of autistic adults—and underscores the urgency of the research agenda we propose.

We synthesize evidence that what is labeled “irritability” in many autistic adults may often be a physiological response to cumulative stress or trauma, or a manifestation of intrinsic neurobiological dysregulation, rather than a primary mood disturbance. By deconstructing the term, critiquing the behavioral checklists that fail to capture the internal states associated with irritability, and mapping emerging therapeutics to their mechanistic targets, we lay out a research agenda that leverages both lived experience and biological precision.

**FIGURE 1:**
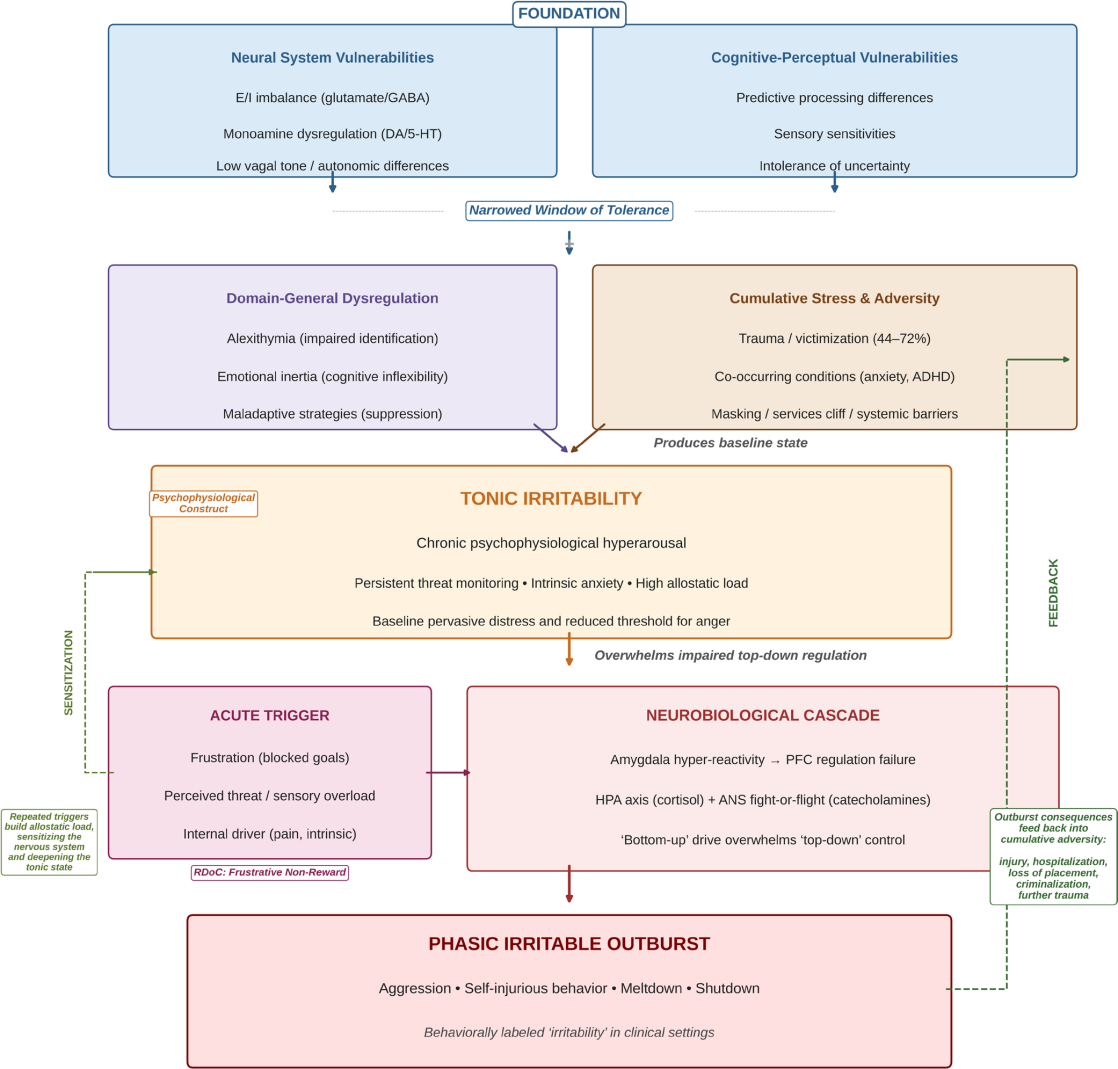
Proposed Conceptual Model of Irritability in Autistic Adults. Underlying neurobiological and cognitive‐perceptual vulnerabilities (Foundation), compounded by domain‐general dysregulation and cumulative stress, produce a baseline state of chronic psychophysiological hyperarousal (Tonic Irritability). Acute triggers then overwhelm impaired top‐down regulatory circuits, initiating a neurobiological cascade that results in a Phasic Irritable Outburst. The model is bidirectional. The dashed arrow on the left (“Sensitization”) represents how repeated or chronic acute triggers themselves build allostatic load and sensitize the nervous system, deepening the tonic state over time. The dashed arrow on the right (“Feedback”) illustrates how outburst consequences (e.g., injury, hospitalization, loss of placement, criminalization, further trauma) feed back into cumulative adversity, perpetuating the cycle. Together, these two pathways convert the model into a dynamic bidirectional system. 5‐HT, serotonin; ADHD, attention‐deficit/hyperactivity disorder; ANS, autonomic nervous system; DA, dopamine; E/I, excitatory/inhibitory; GABA, gamma‐aminobutyric acid; HPA, hypothalamic–pituitary–adrenal; PFC, prefrontal cortex; RDoC, Research Domain Criteria.

To bridge these gaps, we propose a unifying core conceptual model (summarized in Figure 1). Drawing upon recent comprehensive transdiagnostic frameworks of self‐regulation in neurodevelopmental conditions (Iturmendi‐Sabater et al. 2026), our model posits that underlying neurobiological vulnerabilities (e.g., sensory processing differences, excitatory/inhibitory imbalance) and domain‐general dysregulation form the foundation. When compounded by cumulative stress and trauma, this produces a baseline state of chronic psychophysiological hyperarousal and pervasive distress (tonic irritability). Acute environmental triggers (e.g., frustration, threat) then overwhelm impaired top‐down regulation, resulting in the psychophysiological explosion we recognize as a phasic irritable outburst. The model is bidirectional: repeated or chronic acute triggers (e.g., recurrent sensory overload, persistent uncertainty, ongoing social invalidation) themselves build allostatic load and sensitize the nervous system (McEwen 2007), thereby deepening the tonic state—while the consequences of phasic outbursts (e.g., injury, hospitalization, loss of placement, criminalization) feed back into cumulative adversity, further amplifying the underlying vulnerability. The remainder of this review deconstructs this model, critiques current measurement tools, and outlines a roadmap for adult‐focused clinical trials.

A note on language: we use identity‐first terminology (“autistic adults”) throughout this manuscript, reflecting the language preferences expressed by the majority of autistic individuals surveyed across multiple English‐speaking countries (Keating et al. 2023), while acknowledging that preferences are not universal (Amaral 2023). This choice reflects our commitment to centering autistic voices and lived experience throughout the framework we propose.

## 2 The Challenge of Definition: Deconstructing the “Irritability” Construct

As highlighted by our opening question, the conceptual confusion surrounding irritability is widespread. A critical review highlighted profound inconsistencies in how irritability is defined and measured, noting significant overlap with anger and aggression (Toohey and DiGiuseppe 2017). Definitions frequently confound the internal experience of irritability with its causes (e.g., triggers) and consequences (e.g., aggression). This ambiguity is mirrored in the DSM‐5, which handles irritability inconsistently across 15 disorders (Toohey and DiGiuseppe 2017). This terminological ambiguity obscures underlying mechanisms and hinders the development of targeted treatments.

Modern conceptualizations describe irritability as a complex psychophysiological trait or mood state. At its core, it is characterized by a reduced threshold for experiencing anger in response to frustration or annoyance (Leibenluft et al. 2024; Toohey and DiGiuseppe 2017). Rather than viewing it solely as a behavioral outcome or a purely biological reflex, it must be understood as a domain‐general construct at the intersection of affective distress, cognitive processing (e.g., threat appraisal), and autonomic physiological hyperarousal. This aligns with recent meta‐research identifying irritability and heightened baseline arousal as core, transdiagnostic psychological constructs spanning neurodevelopmental conditions (Iturmendi‐Sabater et al. 2026). Furthermore, this conceptualization maps onto the Research Domain Criteria (RDoC) framework's “Frustrative Non‐Reward” construct, where the inability to achieve an expected outcome (often exacerbated by autistic cognitive inflexibility or intolerance of uncertainty) triggers a cascade of negative affect and sympathetic activation (Brotman et al. 2017). While these underlying psychophysiological mechanisms may be shared transdiagnostically, their specific triggers and expressions in autism are profoundly idiosyncratic—reflecting highly individualized, path‐dependent neurodevelopmental trajectories rather than uniform subgroup presentations (Lin et al. 2026).

To achieve clinical precision, irritability must be distinguished from anger (an acute emotional state in response to a perceived provocation) and aggression (the external behavioral manifestation that causes or threatens harm; while often defined by intent, the intent behind aggression in autistic individuals, particularly those with significant communication impairments or intellectual disability, may be absent or impossible to determine) (Lischinsky and Lin 2020). While irritability may often precede aggression, they are conceptually distinct; understanding this psychophysiological mood state is crucial for identifying underlying distress before it escalates into behavior. As introduced in our conceptual model, this dynamic maps onto an influential framework separating tonic irritability (a persistent, chronic grumpy state) (Carlson and Singh 2021) from phasic irritability (frequent, disproportionate outbursts in response to provocation) (Carlson et al. 2023). While the distinction between tonic and phasic irritability is empirically established in the transdiagnostic pediatric literature (Cardinale et al. 2021; Leibenluft et al. 2024), suggesting these two facets may have different neurobiological underpinnings, we adopt it here as a proposed heuristic framework for autistic adults, one that requires urgent empirical validation in this specific population.

It is crucial to note that while irritability is a common pathway to challenging behaviors in autism, it is not the only one. Aggression and SIB are heterogeneous and can occur independent of the affective state of irritability. Some aggression and SIB may be driven by other mechanisms, including automatic reinforcement (occurring independent of social consequences), which accounts for a significant proportion, roughly 25%, of SIB (Hagopian et al. 2015), or intrinsic neurobiological drivers unrelated to frustration or threat (Minshawi et al. 2014; Oliver et al. 2017) or as a manifestation of excited catatonia (Wachtel and Dhossche 2010). Differentiating these pathways is essential, as treatments targeting irritability may be ineffective for behaviors driven by these alternative mechanisms.

This irritability landscape is situated within a broader context of domain‐general self‐regulatory difficulties (Iturmendi‐Sabater et al. 2026), a core challenge for many autistic individuals involving the inability to effectively monitor and modulate emotional experiences (Mazefsky et al. 2013; McDonald et al. 2024). This difficulty is often exacerbated by alexithymia (difficulty identifying and describing internal states), which is particularly prevalent in autistic populations (Poquérusse et al. 2018). To understand how these regulatory breakdowns occur, the Extended Process Model of emotion regulation, which outlines phases of identification, selection, implementation, and monitoring (Gross 2015), offers a useful framework in autistic individuals (Cai and Samson 2025).

Drawing on broader affective neuroscience and overarching reviews of self‐regulation (Iturmendi‐Sabater et al. 2026), we suggest that while emotion dysregulation and emotional inertia (a resistance to emotional change) are transdiagnostic phenomena, their presentation in autism is uniquely magnified by underlying sensory processing differences, social cognitive demands, and high rates of alexithymia (Beck et al. 2020; Mazefsky et al. 2013; Nuske et al. 2024). Extrapolating to autistic adults, breakdowns often occur in the initial identification phase of the Extended Process Model (Gross 2015). When autistic individuals struggle to interpret their own physiological arousal, whether driven by sensory overload or uncertainty (Chen et al. 2024), it prevents the timely deployment of regulatory strategies (South and Rodgers 2017). Furthermore, “emotional inertia,” a resistance to emotional change linked to cognitive inflexibility, may prevent the activation of goals to regulate emotions in the first place in autistic individuals (Cai and Samson 2025). Challenges also arise in the selection phase, as autistic individuals may favor less cognitively taxing strategies (e.g., suppression or avoidance over reappraisal) (Cai and Samson 2025; Dell'Osso et al. 2023). This preference may be an adaptation to chronic high arousal and executive function demands.

These regulatory difficulties lower the threshold for an outburst. When the identification phase fails and arousal escalates unchecked, it can manifest as agitation—a state of excessive motor activity (e.g., restlessness, pacing) and inner tension (Lindenmayer 2000) that often includes irritability as a core component and serves as a direct precursor to aggression. Building on this, we argue that for many autistic adults, what is labeled as “irritability” may more accurately reflect a state of physiological hyperarousal—a chronic sympathetic activation driven by the interplay between a neurobiologically vulnerable system and external stressors (e.g., frustration, threat, recalled trauma) (Brzozowska and Grabowski 2025) or internal drivers (e.g., pain, interoceptive discomfort, intrinsic dysregulation) (Minshawi et al. 2014). This vulnerability should be understood not as an inherent predisposition to aggression, but as a heightened sensitivity and difficulty in regulating physiological arousal (Mazefsky et al. 2013).

## 3 A Spectrum of Experience: How Irritability Manifests Across the Lifespan and Intersecting Identities

Irritability does not present uniformly across the autism spectrum. Its expression and function are shaped by developmental stage, intellectual and communicative abilities, and intersecting identities (Calub et al. 2025) (Table 1).

**TABLE 1:** Heterogeneity of irritability in autistic adults: Manifestations, drivers, and contextual factors.

Domain	Subsection	Key considerations
Support needs	Autistic adults without ID	Irritability often builds internally (rumination, anxiety) before external manifestation Key drivers include Intolerance of Uncertainty (IU), executive function challenges, and violation of rigid expectations (“Black and White” thinking) Masking (camouflaging) is often reported as a significant stressor, though empirical links to mental health outcomes are complex Outbursts can be “implosive” (shutdown) or “explosive” (verbal anger) Triggers include sensory overload, routine disruption, and social misunderstandings (often rooted in bidirectional communication barriers)
	Autistic adults with ID (including profound autism)	Internal irritability is often expressed through immediate challenging behaviors (aggression, SIB, meltdowns) External behavior often serves communication for unmet needs or distress, but may also be driven by automatic reinforcement or intrinsic factors Triggers are frequently physiological (e.g., undiagnosed pain, hunger) or environmental (e.g., sensory overload, abrupt changes) Distinction between irritability, agitation, and aggression is often blurred for observers
Lifespan trajectory	Evolution and persistence	Trajectory diverges: evolves toward chronic hypervigilance/trauma reactivity in some; persists as stable challenging behaviors in others (especially with ID) Adulthood introduces unique stressors (e.g., navigating relationships, parent–child transitions regarding autonomy) The “services cliff” (loss of educational supports) exacerbates irritability and limits options for adults with high support needs Consequences of outbursts escalate significantly with age (e.g., hospitalization, criminal justice involvement)
Intersecting identities	Gender and sex	Autistic women and gender‐diverse individuals are more likely to internalize distress (e.g., self‐injury, anxiety) rather than externalize aggression Masking often obscures irritability until a crisis point (burnout, suicidality) Impact of hormonal fluctuations (e.g., menstrual cycle, menopause) on emotion regulation is often ignored Gender‐diverse individuals face additional minority stress
	Culture and race	Cultural norms (e.g., collectivism, emotional restraint) shape expression and increase pressure to suppress feelings Assessment tools normed on White, Western populations risk misinterpretation Intersectionality compounds vulnerabilities (e.g., Black autistic adults face dual stigma and risk of criminalization of behavior) Poverty exacerbates triggers (e.g., housing instability, lack of therapeutic access)

Abbreviations: ID, intellectual disabilities; IU, intolerance of uncertainty; SIB, self‐injurious behavior.

### 3.1 Across Different Support Needs

It is important to note that due to the lack of large‐scale longitudinal datasets in autistic adults, the lifespan distinctions outlined below and in Table 1 are drawn primarily from clinical synthesis, qualitative reports, and extrapolation from pediatric data. This divergence is clear when comparing autistic adults with and without intellectual disability (ID), particularly adults with profound autism (defined here in alignment with the Lancet Commission as individuals with an IQ < 50, minimal to no verbal communication, and a need for lifelong, 24‐h care (Lord et al. 2022)). Irritability is fundamentally an internal, psychophysiological experience for all autistic individuals. We hypothesize that this internal experience is present across the spectrum, even when profound communication impairments limit its expression strictly to observable behavioral channels. However, while the underlying driver of irritability for both groups is often nervous system hyperarousal, the function and manifestation of the resulting behavior may differ.

For autistic adults without ID, irritability often builds internally before becoming externally visible. It can manifest as rumination, frustration with executive functioning challenges, or pervasive anxiety (Spain et al. 2020). A key driver of irritability in this group is the intolerance of uncertainty; this profound discomfort with ambiguity can transform minor environmental unpredictability into a perceived threat (Wigham et al. 2015). To manage this, individuals may adopt rigid or Black‐and‐White thinking styles (Stark et al. 2021); when these rigid expectations are violated, the distress can amplify irritability. An underrecognized contributor to irritability in autistic adults without ID is pain and interoceptive discomfort. Growing evidence indicates that interoceptive differences are present across the autism spectrum (Klein et al. 2025), and atypical pain processing (including allodynia, paradoxical heat sensation, and altered thermal thresholds) (Bogdanova et al. 2022) may produce chronic low‐grade somatic discomfort that persistently elevates baseline arousal. In autistic adults without ID, articulate verbal accounts of distress can mask this somatic substrate, particularly when co‐occurring alexithymia (Poquérusse et al. 2018) (discussed further in the Measurement section) further blunts internal‐state awareness. Clinicians attuned only to cognitive and social dimensions of irritability may miss pain and interoceptive load as drivers. Beyond these internal drivers, the cognitive and emotional effort of “masking” (camouflaging autistic traits) is often reported qualitatively as a significant, energy‐depleting stressor that may also lower the threshold for irritability (Cage et al. 2018), resulting in outbursts that can be “implosive” (shutdowns) or “explosive” (verbal anger) (Raymaker et al. 2020). We return to the complexity of the camouflaging–mental health relationship in the Measurement section. The aggression associated with irritability in autistic adults without ID is typically a reactive response to feeling overwhelmed, misunderstood, or invalidated, rather than a primary feature (Phung et al. 2021). Frustrations engendered by sensory overload, a disruption to routine (which violates predictability), social misunderstandings, or the exhaustion of masking act as potent triggers (Cage et al. 2018; DuBois et al. 2017). These social misunderstandings are often rooted in bidirectional difficulties in understanding perspectives between autistic and non‐autistic individuals, rather than a one‐sided autistic deficit (Livingston et al. 2025; Milton 2012). The chronic stress of these interactional barriers may activate memories of past social victimization and trauma (Hoover and Kaufman 2018).

Conversely, for autistic adults with ID (including profound autism), communication impairments mean their internal experience of irritability can be expressed through more immediate challenging behaviors, including aggression, SIB, and intense meltdowns (McClintock et al. 2003). Here, the distinction between irritability, agitation, and aggression often blurs, as the external behavior might be the only observable sign of the underlying affective state. While these behaviors frequently serve as a primary method of communication (Edelson 2022; Totsika et al. 2008), it is a critical misconception to assume all challenging behavior in this population is communicative or intentional. Doing so can lead to ineffective interventions that endlessly search for external triggers while ignoring intrinsic drivers (Lutz 2017). Triggers are frequently tied to immediate physiological, environmental or intrinsic drivers. For example, undiagnosed physical pain from gastrointestinal issues (Madra et al. 2020), dental pain, chronic infections, and headaches/migraines (Edelson 2022) can be critical triggers. Sensory overload, abrupt changes to routine, and the inability to communicate basic needs like hunger or thirst are also powerful catalysts (Edelson 2022). Furthermore, severe challenging behaviors in this population may also reflect autistic catatonia, a distinct neuropsychiatric syndrome requiring specific assessment (Wachtel 2019). In this context, aggressive behavior and SIB often serve critical functions: whether as a desperate communication of an urgent, unmet need, or as an attempt to regulate internal states (e.g., managing sensory overload or attenuating discomfort) independent of the social environment (automatic reinforcement).

### 3.2 The Lifespan Trajectory: Evolution and Persistence

The nature of irritability transforms across the lifespan, yet this evolution is rarely tracked by longitudinal research. In childhood, it often presents as tantrums and physical aggression (Mazefsky et al. 2018). The trajectory then diverges based on support needs. Notably, a recent meta‐analysis suggested a trend where the magnitude of difference in emotion dysregulation severity between autistic and neurotypical groups was greater in older samples (McDonald et al. 2024), underscoring the persistence and potential escalation of these challenges into adulthood. For autistic individuals without ID, with the transition to adolescence, hormonal changes can exacerbate emotional dysregulation, while mounting social pressures and a painful awareness of being different can fuel frustration and the accumulation of trauma (Picci and Scherf 2015). By adulthood, these experiences coalesce; what began as situational frustration may evolve into chronic hypervigilance and trauma‐based reactivity. Furthermore, adulthood introduces unique interpersonal stressors, such as navigating romantic relationships or managing the complex transition of the parent–child relationship as both parties age (Stapley et al. 2021). Difficulties navigating evolving expectations regarding autonomy and dependence can become significant sources of friction and contribute to irritability, even when parents are not serving in a formal caregiver role (Valdez et al. 2013). Adults often develop exhausting coping mechanisms that may temporarily mask the overt expression of irritability but amplify internal distress, leading to more severe eventual episodes (Pearson and Rose 2021).

Conversely, for autistic adults with ID, including profound autism, irritability and associated challenging behaviors often persist throughout the lifespan. Longitudinal studies indicate high stability of behaviors; for example, one study found that 84% of individuals with developmental disabilities (including autism) continued to exhibit SIB over a 20‐year period (Taylor et al. 2011). The transition to adulthood is particularly perilous; as individuals face the aforementioned “services cliff” (Westover 2024), the abrupt loss of structured educational settings and stabilizing routines often exacerbates irritability and limits service options. Furthermore, reliance on aging parents who may be experiencing declining health creates a precarious situation, increasing the risk of crisis and institutionalization.

Critically, the consequences of these overt expressions amplify with age across the spectrum. As individuals grow bigger and stronger, the potential for injury and damage increases. What once resulted in timeouts now leads to hospitalization, criminal justice involvement, or the loss of housing and support services. This creates a vicious cycle where the fear of an irritable episode itself may become a potent trigger for the very hyperarousal that causes it.

This cycle warrants closer attention because the consequences of irritable outbursts are not merely downstream outcomes—they are themselves traumatogenic events that deepen the tonic state. Restraint, seclusion, repeated emergency department visits and psychiatric admissions, the loss of community placement and supportive housing, and increasingly the criminalization of behavior driven by sensory or stress‐driven dysregulation, all add to the very burden of cumulative adversity from which tonic irritability emerges. For autistic adults with ID, these consequences are compounded by the limited recognition of behavior as communication and the disproportionate use of restrictive interventions (Lutz 2017). For autistic adults without ID, the same dynamics often manifest more subtly through invalidation, social exclusion, employment loss, and contact with the criminal justice system following meltdowns that are misread as willful aggression (Phung et al. 2021). The bidirectional amplification depicted in Figure 1—trigger‐driven sensitization on one side, outburst‐driven adversity accumulation on the other—thus constitutes a self‐perpetuating clinical trap. Recognizing it as such reframes the goals of intervention: alongside reducing immediate behavioral manifestations, clinicians and systems must explicitly aim to interrupt the cycle by minimizing iatrogenic and systemic consequences (e.g., trauma‐informed crisis response, alternatives to restraint, reduced criminalization) that would otherwise feed back into the tonic state.

### 3.3 Intersecting Identities: The Influence of Gender, Culture, and Race

An individual's intersecting identities, encompassing factors such as gender, race, culture, and socioeconomic status, significantly shape the experience of irritability. These factors influence how irritability is experienced internally and how it is interpreted and responded to by others (e.g., via stereotypes). Furthermore, these identities shape an individual's exposure to systemic stressors, such as discrimination and lack of access to care, which in turn can significantly exacerbate irritability.

Empirically, clinical presentations of irritability can be shaped by sex and gender. For instance, research demonstrates that autistic women (Song et al. 2025) and gender‐diverse individuals (Cooper et al. 2022) may be more likely to internalize distress, expressing it through self‐injury, eating dysregulation, or severe anxiety rather than outward aggression (Antezana et al. 2019). This internalization, coupled with masking, means that irritability may be frequently missed by clinicians and families until it reaches a crisis point of burnout, self‐harm, or suicidal ideation or behaviors (Bargiela et al. 2016). The impact of hormonal fluctuations (e.g., menstrual cycle, pregnancy, hormonal medications, and menopause) on emotion regulation in autistic women is an often ignored factor in assessment and treatment (Groenman et al. 2022). Gender‐diverse autistic individuals may face an additional layer of minority stress and dysphoria‐related triggers that can compound baseline vulnerabilities to irritability (Cooper et al. 2022).

Conversely, the compounding effects of other intersectional identities represent highly plausible but currently understudied mechanisms. Cultural (Ma et al. 2023) and racial (Benevides et al. 2024) factors theoretically shape how irritability is expressed, interpreted, and managed. In collectivist cultures that value group harmony and emotional restraint (Tsai and Lu 2018), for example, autistic adults may face intense pressure to suppress their feelings, leading to more severe internalized presentations (Ma et al. 2023). Current assessment tools, normed predominantly on White, Western populations, risk both pathologizing culturally sanctioned emotional expressions and missing genuine distress that is masked by cultural coping styles. The intersection of multiple marginalized identities creates compound vulnerabilities. We hypothesize that Black autistic adults, who face the dual stigma of racial stereotypes about aggression and disability‐related behavioral differences, experience heightened baseline threat responses due to systemic discrimination. This can lead to disproportionate police involvement and the criminalization of irritability‐driven behaviors (McCauley et al. 2018), though empirical validation of this specific physiological pathway in adult autism research remains scarce. For those experiencing poverty, triggers multiply in the face of sensory‐assaultive housing, food insecurity, and an inability to afford therapeutic support. This lack of culturally responsive care, combined with the absence of research samples to represent this diversity, means interventions are often ineffective for those facing multiple layers of oppression (Hall et al. 2020).

## 4 The Measurement Crisis: Why Current Tools Fail Autistic Adults

The heterogeneity of irritability in autistic adults creates a fundamental measurement crisis. The field's reliance on tools that were not designed for or validated in this diverse population means we often measure behavioral consequences rather than the core affective experience. This crisis is two‐fold: flawed instruments and methodologically weak studies.

### 4.1 A Flawed Toolkit: Critiquing Existing Measures

The primary tools used to assess irritability in autism research and clinical practice are poorly suited for the task in adults. This reliance on questionnaires is symptomatic of a field‐wide methodological bottleneck. A recent large‐scale meta‐summary of self‐regulation measurement across neurodevelopmental conditions revealed that over 72% of measurement instances rely solely on questionnaires, with a staggering dependence on parent/caregiver proxy‐reports (used in 62% of autism studies and 100% of studies of intellectual disability) (Iturmendi‐Sabater et al. 2026). Consequently, the evidence base for FDA‐approved medications rests almost entirely on caregiver‐rated scales like the Aberrant Behavior Checklist‐Irritability subscale (ABC‐I) (Aman et al. 1985). The ABC‐I captures a broad construct of behavioral dysregulation (including aggression, SIB, and tantrums) rather than the specific affective mood of irritability (Evans et al. 2024). By conflating the internal affective state (irritability) with its behavioral consequences (e.g., aggression), the ABC‐I and similar scales fail to accurately measure the core construct (Toohey and DiGiuseppe 2017). This creates a dangerous conflation: drugs are approved for “irritability” based on trials that primarily measured reduced disruptive behaviors, which may occur (e.g., via sedation) without improvement in the underlying affective distress. Newer caregiver‐report scales like the Emotion Dysregulation Inventory (EDI), developed specifically for ASD, offer a more nuanced approach by separating “Reactivity” (akin to irritability) from “Dysphoria,” but their use is not yet widespread as the ABC‐I (Mazefsky et al. 2018).

For autistic adults without ID, while subjective scales have limitations, they should not be discarded. Instruments specifically designed to capture the affective core of irritability with minimal behavioral conflation, such as the Affective Reactivity Index (ARI) (Stringaris et al. 2012) or the Brief Irritability Test (BITe) (Holtzman et al. 2015), offer access to subjective experience and hold significant value (Saatchi et al. 2023). Furthermore, recent research has administered the BITe to a large neurotypical adult sample in the United States, providing valuable potential population norms for irritability across the adult lifespan (Perlis et al. 2024). Rather than replacing self‐report, the field must move toward a multi‐modal assessment strategy, as current standard instruments often overlook the dynamic interdependence of emotional, cognitive, and physiological components (Iturmendi‐Sabater et al. 2026). Future research should prioritize the qualitative validation and adaptation of these subjective scales for autistic adults, such as adjusting language to accommodate literal interpretation styles or utilizing visual and concrete scaling. When adapted appropriately, these scales provide the crucial subjective psychological context needed to interpret emerging objective physiological measures. Currently, however, the unadapted use of these scales can be compromised by core autistic traits (Beck et al. 2020). As previously noted, alexithymia presents a direct barrier (Poquérusse et al. 2018), preventing individuals from recognizing and reporting escalating arousal until a crisis point is reached (South and Rodgers 2017). A tendency toward literal interpretation can lead to misunderstanding of questionnaire items, and masking itself can lead to reports that reflect a constructed persona rather than a true internal state (Pearson and Rose 2021). However, the empirical relationship between camouflaging and mental health is more complex than a simple stress‐accumulation account, with implications for how we measure irritability in this group. A recent two‐year longitudinal study found that higher baseline camouflaging was associated with a small but significant decrease in mental health difficulties over time, contrary to predictions (van der Putten et al. 2025). Several non‐mutually‐exclusive interpretations may reconcile this with the well‐replicated cross‐sectional links between camouflaging and distress (Arnold et al. 2026): (i) selection effects, whereby individuals with greater intrinsic regulatory capacity may both camouflage more and recover better; (ii) heterogeneity within camouflaging itself—the assimilation subtype (suppressing one's identity to fit in) appears most strongly linked to distress, whereas compensation strategies may be context‐adaptive; and (iii) the possibility that successful, voluntary camouflaging in supportive contexts is materially different from compulsory or burnout‐producing camouflaging in hostile ones. This complexity reinforces, rather than undermines, the central argument: behavioral measures alone cannot capture the affective and physiological cost of regulatory effort, and only mechanism‐informed assessment can disentangle adaptive from harmful expressions of the same observable behavior.

Finally, clinician‐rated tools like the Clinician Affective Reactivity Index (CL‐ARI) (Haller et al. 2020) or the Clinical Global Impression (CGI) scales provide a standardized framework. However, they are based on brief clinical snapshots where behavior may be atypical. A significant concern is “diagnostic overshadowing,” the tendency to attribute all challenging behaviors to autism itself, causing clinicians to miss underlying drivers like pain, anxiety, or trauma, or distinct co‐occurring psychiatric conditions (e.g., bipolar disorder, major depression, ADHD) (Edelson 2022; Kerns et al. 2015), or related conditions such as catatonia (Wachtel 2019).

### 4.2 Compounding the Crisis: Methodological Flaws in Adult Research

The few studies on irritability that include autistic adults are undermined by methodological flaws (Choi et al. 2024). A critical issue is sample homogeneity, as most studies recruit from specialized clinics, capturing only a fraction of the autistic population and systematically excluding adults with profound autism (Brugha et al. 2016). Further, study designs are often inadequate: researchers use pediatric‐focused measures with adults, short follow‐up periods prevent assessment of long‐term efficacy, and a failure to control for polypharmacy makes it nearly impossible to isolate specific treatment effects (Jobski et al. 2017). Exclusion criteria often eliminate individuals with the most severe irritability due to safety concerns as well as practical and regulatory barriers (e.g., recruitment challenges, difficulties obtaining informed consent for those lacking capacity, and stringent research ethics requirements), paradoxically shutting out the very population that most needs intervention. Furthermore, the traditional case–control paradigms used in these trials inherently rely on group averages. Recent theoretical frameworks highlight that the autistic brain is fundamentally characterized by idiosyncrasy—deeply individualized, stable physiological and cognitive patterns that persist after accounting for broad demographic variables, reflecting heavy‐tailed, non‐Gaussian distributions of neurodevelopmental outcomes (Lin et al. 2026). By averaging across highly heterogeneous adult samples, traditional irritability trials risk blurring these meaningful, person‐specific physiological signals into statistical noise.

## 5 The Measurement Revolution: The Need for Objective, Physiologically‐Informed Tools

Given the limitations of subjective scales and the physiological nature of the irritability response, a paradigm shift in measurement is imperative. The integration of wearable biosensors offers an opportunity to objectively quantify the internal states preceding and constituting an irritable episode. This is crucial given emerging evidence of emotional discordance in autistic children, where internal physiological arousal does not align with external communicative expressions (Finkel et al. 2024). This discordance, which may be linked to alexithymia (Poquérusse et al. 2018), means that an autistic person can experience significant internal distress invisible to observers, rendering subjective and observational reports alone insufficient.

Fortunately, recent research, although almost exclusively pediatric, has demonstrated the feasibility and validity of using wearable biosensors with autistic individuals (Nuske et al. 2022). Physiological signals, including heart rate (Goodwin et al. 2019; Nuske et al. 2019) and electrodermal activity (EDA; a direct measure of sympathetic arousal) (Goodwin et al. 2019), can predict the onset of challenging behaviors, on average 60–80 s before the behavior occurs. More recently, machine learning analysis of biosensor data (cardiovascular, EDA, and motion signals) predicted aggressive behavior 3 min before its onset with high accuracy in inpatient autistic youth (Imbiriba et al. 2023). This extended early warning signal significantly enhances the window for proactive intervention. Beyond prediction, physiological data serves as powerful, objective outcome measures for clinical trials. Changes in heart rate predict intervention effectiveness (Emezie et al. 2024). Further, heart rate variability (HRV) provides a biomarker for self‐regulation capacity (Cai et al. 2019; Reisinger et al. 2024). Actigraphy can quantify sleep disturbances, a major contributor to irritability (Bangerter et al. 2020), and motor agitation (Rad et al. 2025). This technology is already being translated into clinical tools. Digital mental health applications, such as the KeepCalm app, are being developed to integrate real‐time physiological data from wearables to provide just‐in‐time, individualized strategy recommendations to teachers and caregivers, with protocols for pilot RCTs already established (Palermo et al. 2023).

Despite this promise, we must confront a stark reality: virtually all of this evidence comes from pediatric samples, and adult validation is currently absent. Given that naturalistic behavioral observations and objective physiological/cognitive tasks currently account for less than 30% of self‐regulation measurements in neurodevelopmental research (Iturmendi‐Sabater et al. 2026), addressing this gap is paramount. Such dense, longitudinal sampling of intraindividual physiological data is precisely what is required to capture personalized biosignatures, circumventing the limitations of traditional group averages (Lin et al. 2026). Translating wearable biosensor technology to autistic adults, however, presents unique scientific and practical challenges. Physiologically, decades of chronic stress, co‐occurring medical conditions, and polypharmacy (particularly the autonomic‐blunting effects of antipsychotics or beta‐blockers) may fundamentally alter baseline physiological signatures (e.g., dampening heart rate variability or electrodermal reactivity), potentially invalidating predictive algorithms trained on pediatric samples (Alvares et al. 2016). Practically, significant barriers remain for adult implementation, including sensory tolerability, high costs, compliance, and the computational complexity of interpreting noisy data (e.g., distinguishing motor stereotypies from agitation‐related motion) (Taj‐Eldin et al. 2018).

Integrating continuous physiological signals with nuanced self‐report scales could yield a “digital phenotype” of irritability, enabling personalized interventions. However, this must be pursued with caution. Wearable sensors capture intimate data, raising universal privacy and data security concerns. Importantly, as with any medical monitoring technology, its use must always be voluntary and based on informed consent (Kirkham and Greenhalgh 2015). Furthermore, individuals with profound autism may not tolerate wearables. For adults unable to provide informed consent, the deployment of this technology requires rigorous safeguards and careful ethical deliberation involving families/guardians and ethics boards. This is necessary to balance potential therapeutic benefits (e.g., preventing severe injury) with the protection of bodily autonomy and data privacy. Therefore, co‐designing research protocols with autistic people and their families/caregivers, transparent consent processes, clear data‐sharing plans, and user control over monitoring are essential ethical prerequisites.

## 6 A Deeper Look: The Neurobiology of Stress, Threat, and Irritability in Autism

Having established that irritability in autistic adults often reflects physiological hyperarousal, we now examine the specific neural circuits and biological systems that create this vulnerability and mediate the cascade from trigger to outburst. A leading translational model posits that irritability arises from aberrant responses to two key environmental cues: frustration (blocked goal attainment) and threat (Leibenluft et al. 2024).

### 6.1 A Vulnerable Nervous System Meets a Hostile World

The autistic nervous system can be conceptualized as having a narrower window of the optimal state of tolerance where information can be processed effectively (Corrigan et al. 2011). This is due in part to baseline differences in physiological regulation, including evidence of low vagal tone, meaning the parasympathetic (“rest‐and‐digest”) system struggles to efficiently counteract stress responses (Cai et al. 2019). Furthermore, a foundational theory in autism neurobiology posits an imbalance between excitatory (glutamate) and inhibitory (GABA) neurotransmission (E/I imbalance), often favoring excitation (Rubenstein and Merzenich 2003). This imbalance is thought to contribute to neural hyper‐reactivity and sensory sensitivities. Dysregulation in monoamine systems, specifically dopamine (DA) and serotonin (5‐HT), further contributes to this vulnerability (Martin et al. 2025). These neurotransmitters are critical modulators of irritability (Narvaes and Martins de Almeida 2014) and are the primary targets of currently approved pharmacological treatments. Recent translational models suggest an intricate interplay in autism where 5‐HT acts as a “brake” and DA as an “accelerator” for aggression. Specifically, diminished 5‐HT function may increase aggression in response to perceived social threats (heightened threat sensitivity), while heightened DA signaling might drive aggression in response to the disruption of routines or rigid expectations (Martin et al. 2025). Together, these factors lower the threshold for stress‐induced hyperarousal.

This baseline physiological vulnerability is often amplified by co‐occurring psychiatric conditions (such as anxiety, depression, and attention‐deficit/hyperactivity disorder) which are common (Lai et al. 2019) and linked to irritability (Manter et al. 2025). Emerging perspectives suggest that emotion dysregulation may serve as a critical intermediate step in the illness trajectory from core autistic traits (e.g., perseveration) to the development of these mood and anxiety disorders (Dell'Osso et al. 2023). While conditions such as bipolar disorder can present with distinct episodes of elevated mood and irritability (Vasconcelos‐Moreno et al. 2025), we focus here on integrating the chronic distress associated with these conditions into a core framework. Of particular importance is “intrinsic anxiety” in autism—a pervasive arousal stemming directly from core autistic features such as sensory sensitivities, intolerance of uncertainty, and the cognitive demands of navigating unaccommodating environments (South and Rodgers 2017; Stark et al. 2021).

At a cognitive level, this intrinsic anxiety can be driven by differences in predictive processing, whereby attenuated top‐down predictions about the environment lead to an increased reliance on bottom‐up sensory evidence (Pellicano and Burr 2012). This results in larger “prediction errors”—the difference between what is expected and what occurs (Pellicano and Burr 2012). These neural computational differences make the world feel inherently unpredictable, biologically underpinning the profound intolerance of uncertainty discussed earlier (Wigham et al. 2015). When this high need for predictability is inevitably blocked by real‐world ambiguity, it generates significant frustration and forces the nervous system into persistent threat monitoring, locking the individual into a chronic state of hypervigilance (South and Rodgers 2017; Stark et al. 2021). Furthermore, differences in perception and information processing may cause autistic individuals to perceive adverse experiences as more traumatic and lead to ineffective coping strategies (Takeda et al. 2024). This is compounded by a distinct physiological response to stress; autistic individuals often exhibit atypical autonomic regulation, characterized by heightened baseline arousal yet blunted phasic responses to acute stressors, indicating “response saturation” (Takeda et al. 2024). This inherent vulnerability is then catastrophically compounded by high rates of victimization (a 44% pooled prevalence reported in a meta‐analysis (Trundle et al. 2023)) and a lack of environmental accommodation. The impact is profound across the spectrum: in a survey of 687 autistic adults, 72% reported at least one interpersonal trauma and 44% met criteria for PTSD (Reuben et al. 2021). Similarly, victimization is prevalent among autistic adults with intellectual disabilities, with a study reporting 34% experiencing violence and 17% experiencing sexual abuse (Kildahl and Helverschou 2024). These accumulated adverse experiences—spanning chronic social victimization (e.g., bullying, rejection, invalidation), abuse, and violence—are a significant contributing factor leading to post‐traumatic stress symptoms and chronic hyperarousal (Peterson et al. 2019; Takeda et al. 2024). The relationship between irritability and adverse experiences is likely bidirectional. While trauma fuels hyperarousal, pre‐existing irritability and challenging behaviors may also increase the risk of social isolation, victimization, neglect, or restrictive interventions, creating a vicious cycle that amplifies distress.

### 6.2 The Neurobiological Cascade: From Trigger to Outburst

What is often labeled an “irritable outburst” can be mechanistically understood as a physiological stress or threat response—often akin to a somatic flashback. This cascade often begins when a present‐day frustration or uncertainty (e.g., social misunderstanding, change in plans, sensory ambiguity) acts as a trigger, activating sensory‐laden memories of past adverse experiences (Peterson et al. 2019). However, as noted earlier, the cascade may also be initiated by internal states or intrinsic neurobiological dysregulation. Regardless of the source, this leads to hyper‐reactivity in the amygdala, the brain's threat‐detection center. The perception of threat in these ambiguous situations may be amplified by atypical integration involving the insula cortex, which is critical for integrating interoceptive (internal bodily states) and sensory information (Fermin et al. 2022). A failure to accurately process and interpret these internal signals contributes to emotional confusion and subsequent hyperarousal. Modulation of this alarm response relies on the prefrontal cortex (PFC) (Etkin et al. 2015). However, autistic individuals with disruptive behaviors show reduced functional connectivity between the amygdala and the ventrolateral PFC during threat processing (Ibrahim et al. 2019), impairing this regulatory capacity. This impairment, coupled with broader network disruptions, leads to difficulties with habituation and an enhanced perception of threat (South and Rodgers 2017). Consequently, the regulatory circuit fails, leaving the amygdala's alarm unchecked, consistent with a classic model of “bottom‐up” drive overwhelming “top‐down” control (Siever 2008).

This unopposed amygdala signal activates a core aggression circuit, including the ventromedial hypothalamus (VMHvl) and downstream projections to brainstem areas like the periaqueductal gray (PAG), which controls innate aggressive actions like biting and vocalizations (Lischinsky and Lin 2020). Simultaneously, the activation triggers the hypothalamic–pituitary–adrenal (HPA) axis, flooding the body with stress hormones (e.g., cortisol), and the Autonomic Nervous System (ANS), initiating a full sympathetic “fight‐or‐flight” response (hyperarousal) (Brzozowska and Grabowski 2025). This state is characterized by increased heart rate, blood pressure, and the release of catecholamines (e.g., norepinephrine); chronic activation of this system contributes to long‐term physiological strain (Carnovale et al. 2022). With no immediate physical threat to fight or flee, this intense internal alarm is discharged through external behaviors: shouting, aggression, or shutdown—actions that constitute an outburst and are often collectively, though imprecisely, labeled as “irritability” in clinical settings.

These multi‐level neurobiological vulnerabilities map effectively onto the distinction between tonic and phasic irritability. From a dynamical systems perspective, autistic neurodevelopment generates a landscape characterized by deep, individualized attractors and altered stochasticity (Lin et al. 2026). Within this framework, the chronic state of sympathetic hyperarousal—driven by persistent sensory prediction errors, high allostatic load, and intrinsic anxiety—manifests as tonic irritability. Against this primed backdrop, an acute trigger initiates the unchecked amygdala‐to‐hypothalamus cascade described above. In dynamical system terms, this forces a sudden, stochastic “jump” across the neurobiological landscape, resulting in the explosive shift to phasic irritability. In simpler terms, chronic hyperarousal creates a system primed for sudden, explosive shifts.

Understanding this pathway reframes irritability not as a volitional act of anger, but as the behavioral manifestation of a nervous system overwhelmed by accumulated stress or perceived threat. This shifts the focus from “anger management” to stress reduction and nervous system regulation, necessitating approaches that account for the individual's history of trauma and adverse experiences. These circuit insights point to pharmacological and psychosocial levers described next.

## 7 The Next Therapeutic Frontier: Beyond Antipsychotics (Table 2)

**TABLE 2:** Promising therapeutic targets and interventions for irritability in autistic adults.

Target/mechanism	Intervention class	Specific agents/approaches	Evidentiary base	Rationale and evidence notes
Antipsychotics	Atypical Antipsychotics (FDA Approved)	Risperidone (Dinnissen et al. 2015), Aripiprazole (Farmer and Aman 2011)	Pediatric RCTs	Established efficacy for irritability in pediatric RCTs; significant metabolic side effects limit long‐term utility in adults
	Novel Antipsychotics (Off‐Label/Investigational)	Brexpiprazole (Stroud et al. 2025), Cariprazine (Citrome et al. 2024), Pimavanserin (Soogrim et al. 2021), Lumateperone (Jawad et al. 2022)	Transdiagnostic Extrapolation	Agents with partial dopamine agonism or unique serotonergic profiles; potentially favorable metabolic profiles; Require RCTs in autistic adults
	Long‐Acting Injectables (LAIs) (Off‐Label)	Aripiprazole Lauroxil (example) (Sun et al. 2025)	Transdiagnostic/Pediatric Observational	Potential utility for severe, persistent irritability where oral adherence is a challenge; Requires study
E/I Balance (Glutamate/GABA)	NMDA Receptor Modulators	Memantine (Ghaleiha et al. 2013), Amantadine (Mohammadi et al. 2013)	Pediatric RCTs	Target E/I imbalance; promising adjuncts in pediatric trials
	Rapid‐Acting Glutamatergic Modulators	Ketamine, Esketamine (Krystal et al. 2024; Ralston et al. 2024)	Theoretical Rationale/Sparse Case Reports	Potential to “reset” maladaptive trauma‐related circuits; evidence in autism sparse
	Other Glutamatergic Modulators	Riluzole (Wink et al. 2018), Dextromethorphan/Bupropion (Stahl 2019)	Pediatric Pilot RCT/Transdiagnostic Extrapolation	Mechanisms targeting glutamate homeostasis or multimodal action; warrant investigation
	GABA Agonists	Arbaclofen (GABA‐B) (Bahji et al. 2025; Veenstra‐VanderWeele et al. 2017)	Pediatric RCTs	Demonstrated efficacy in reducing agitation in autistic youth
	Antioxidant/Glutamate Modulator	N‐Acetylcysteine (NAC) (Ghanizadeh and Moghimi‐Sarani 2013; Hardan et al. 2012; Nikoo et al. 2015)	Pediatric RCTs	Modulates glutamate homeostasis; consistent promise in pediatric RCTs
Autonomic/Adrenergic Regulation	Beta‐Blockers	Propranolol (London et al. 2024)	Pilot RCT (Mixed Adult/Adolescent)	Directly targets sympathetic hyperarousal; high doses show promise for severe aggression
	Alpha‐2 Adrenergic Agonists	Clonidine (Kaye et al. 2024), Guanfacine (Scahill et al. 2015)	Pediatric RCTs	Reduce central sympathetic outflow; some evidence for clonidine in reducing irritability
Cholinergic System	Nicotinic Agonists	Nicotine Patch (investigational) (Lewis et al. 2018)	Adult Direct Evidence (Exploratory Pilot Trial)	Pilot trial suggested anti‐irritability effects.
Neuroinflammation	Immunomodulatory Agents	Minocycline (Erickson et al. 2023), Celecoxib (Asadabadi et al. 2013), Pregnenolone (Ayatollahi et al. 2020), Omega‐3 fatty acids (Amminger et al. 2007; Mazahery et al. 2019), Palmitoylethanolamide (Khalaj et al. 2018)	Pediatric RCTs	Target neuroinflammation; pediatric RCTs found adjunctive benefits improving irritability scores compared to risperidone alone
Endocannabinoid System	Phytocannabinoids	CBD (Aran and Cayam Rand 2024; Bar‐Lev Schleider et al. 2019), CBDV (Iannotti et al. 2014)	Mixed Observational Studies/Theoretical Rationale	Anxiolytic and anti‐inflammatory properties; observational studies report benefits, but RCT evidence remains preliminary/mixed
	Synthetic Cannabinoids	Nabilone (Lin et al. 2023)	Adult Direct Evidence (Phase 1 Trial Protocol)	Under investigation in Phase 1 trial for adults with IDD
Anxiety (Serotonergic)	Partial 5‐HT1A Agonists	Buspirone (Ghanizadeh and Ayoobzadehshirazi 2015)	Pediatric RCT	Network meta‐analysis supports high effectiveness as an adjunct for agitation in autistic youth
Neuromodulation	Non‐Invasive Brain Stimulation	rTMS (Ni et al. 2024; Ni et al. 2023; Xiao et al. 2025)	Mixed Pediatric and Adult RCTs	Potential to directly modulate amygdala‐prefrontal circuits; evidence mixed
		tDCS (Singh et al. 2026)	Pediatric RCTs	A meta‐analysis shows behavioral and emotional regulation improvements in autistic children
		ECT (Wachtel et al. 2025)	Clinical Synthesis/Case Series	Strong beneficial evidence for treatment‐resistant, severe, and life‐threatening aggression or SIB
Emotion Regulation and Distress Tolerance	Psychosocial Interventions	Dialectical Behavior Therapy (DBT) (Bemmouna et al. 2025; Weiner et al. 2025)	Adult Direct Evidence (RCTs)	Effective for emotion dysregulation and self‐harm in autistic adults without ID; targets alexithymia
		Mindfulness‐based treatments (MBT) (Simione et al. 2024; Spek et al. 2013)	Adult Direct Evidence (RCTs)	May reduce anxiety by increasing emotional awareness and coping capacity
Intolerance of Uncertainty (IU)		Adapted Cognitive Behavioral Therapy (CBT) (e.g., CUES program) (Rodgers et al. 2023; Rodgers et al. 2017)	Pediatric Feasibility Trials	Targets core mechanism of IU; shows promise in youth, requires urgent study in adults
Environmental Modification and Skill Building		Applied Behavior Analysis (ABA) (Gitimoghaddam et al. 2022; Newcomb and Hagopian 2018)	Pediatric RCTs	Most widely implemented behavioral intervention but extremely limited evidence in adults. Contested: Many caregivers view it as essential while many self‐advocates raise concerns
		Caregiver/Staff Training and Support (Bearss et al. 2015; Choi et al. 2024)	Pediatric RCTs and meta‐analysis	Highly effective in pediatric populations; urgent need to adapt models for adult caregivers and support staff
		Positive Behavior Support (PBS) (Durand et al. 2013; McClean et al. 2005)	Pediatric RCTs and Transdiagnostic Adult IDD Data	Person‐centered approach utilizing behavioral principles; focusing on quality of life and environmental modification

Abbreviations: CBD, cannabidiol; CBDV, cannabidivarin; E/I, excitatory/inhibitory; ECT, electroconvulsive therapy; GABA, gamma‐aminobutyric acid; ID, intellectual disabilities; IDD, intellectual and developme ntal disabilities; NMDA, N‐methyl‐D‐aspartate; RCT, randomized controlled trial; rTMS, repetitive transcranial magnetic stimulation; SIB, self‐injurious behavior; tDCS, transcranial direct current stimulation.

The treatment paradigm for irritability in autistic adults has been stagnant for two decades, relying heavily on atypical antipsychotics, risperidone (Dinnissen et al. 2015), and aripiprazole (Farmer and Aman 2011). Although meta‐analyses confirm their efficacy (Bahji et al. 2025; Choi et al. 2024), their utility is limited by significant side effects (Dinnissen et al. 2015; Farmer and Aman 2011). These adverse effects drastically increase the risk of metabolic syndrome (e.g., obesity, diabetes, dyslipidemia) (Goltz et al. 2019), which is particularly devastating given that autistic adults already face significant disparities in cardiometabolic health and premature mortality (Bishop et al. 2023). The field must urgently pivot to novel, mechanism‐based treatments (Shamabadi et al. 2024). In the absence of evidence‐based options, off‐label prescribing of other antipsychotics, mood stabilizers, and anti‐epileptics is common in clinical practice (Manter et al. 2025), despite limited evidence for their efficacy in this population (Gupta and Gupta 2024). Many agents studied for broad “irritability” have mechanisms relevant to the model of stress/threat‐induced hyperarousal, offering ways to dampen the physiological stress response cascade (Sippel et al. 2024).

A glaring lack of RCTs in autistic adults exists for newer antipsychotics with more favorable metabolic profiles. These include agents with partial dopamine agonism such as brexpiprazole (Stroud et al. 2025) and cariprazine (Citrome et al. 2024), as well as pimavanserin (a selective serotonin 5‐HT2A inverse agonist that avoids dopamine D2 receptor blockade) (Soogrim et al. 2021) and lumateperone (a multi‐target agent acting on serotonin, dopamine, and glutamate) (Jawad et al. 2022), which are associated with irritability in other mental health conditions. Furthermore, long‐acting injectable formulations (e.g., Aripiprazole lauroxil (Sun et al. 2025)) warrant study for adults with severe, persistent irritability where oral adherence is a challenge (due to refusal, cognitive impairment, or logistical barriers).

Another key strategy involves targeting the excitatory/inhibitory (E/I) imbalance (Rubenstein and Merzenich 2003). NMDA receptor modulators like memantine (a low‐affinity NMDA antagonist) (Ghaleiha et al. 2013) and amantadine (Mohammadi et al. 2013) have shown promise as adjuncts in pediatric trials. Ketamine and esketamine are other NMDA receptor antagonists that promote synaptogenesis and could potentially “reset” maladaptive trauma‐related circuits (Krystal et al. 2024). Evidence in autism, however, remains sparse (Ralston et al. 2024). Other glutamatergic modulators, such as riluzole (Wink et al. 2018), and multimodal agents with NMDA antagonism (e.g., dextromethorphan/bupropion (Stahl 2019)) also warrant investigation. Further targeting E/I balance, the GABA‐B receptor agonist Arbaclofen, which enhances inhibitory signaling, demonstrated efficacy in reducing agitation in autistic youth (Bahji et al. 2025; Veenstra‐VanderWeele et al. 2017). N‐Acetylcysteine (NAC), a precursor to the master antioxidant glutathione and a modulator of glutamate homeostasis, has shown consistent promise in multiple RCTs in autistic children, both as monotherapy (Hardan et al. 2012) and adjunctive treatment (Ghanizadeh and Moghimi‐Sarani 2013; Nikoo et al. 2015).

Another frontier is autonomic and adrenergic regulation. Given the central role of sympathetic hyperarousal and catecholamine release in the physiological stress response cascade (Carnovale et al. 2022; Takeda et al. 2024), modulating the ANS is a key therapeutic strategy. The non‐selective beta‐blocker propranolol directly targets the physical manifestations of sympathetic hyperarousal (e.g., increased heart rate) by blocking the effects of adrenaline. High‐dose propranolol has shown promise for severe aggression in pilot studies (London et al. 2024), though close monitoring for cardiovascular side effects (e.g., hypotension, bradycardia) is required. Alpha‐2 adrenergic agonists like clonidine and guanfacine act centrally to reduce sympathetic outflow from the brainstem, thereby promoting calmness. There is some pediatric evidence for clonidine in reducing irritability (Kaye et al. 2024) and guanfacine in improving impulsivity (Scahill et al. 2015). The cholinergic system, which modulates attention and arousal, also appears to be an under‐explored target, following a pilot open‐label trial of a nicotine patch that showed potent anti‐irritability effects (Lewis et al. 2018).

Targeting neuroinflammation (Arteaga‐Henríquez et al. 2023) is a rapidly emerging field. These agents aim to reduce the activity of pro‐inflammatory cells (like microglia) and signaling molecules (cytokines) in the brain (Arteaga‐Henríquez et al. 2023). Pediatric RCTs have found benefits for diverse agents used adjunctively with risperidone, including the antibiotic minocycline (Erickson et al. 2023), the NSAID celecoxib (Asadabadi et al. 2013), and the neurosteroid pregnenolone (Ayatollahi et al. 2020). Network meta‐analysis (Bahji et al. 2025) also supports the efficacy of dietary supplements with anti‐inflammatory properties, such as omega‐3 fatty acids (polyunsaturated fats) (Amminger et al. 2007; Mazahery et al. 2019), and adjunctive palmitoylethanolamide (an endogenous fatty acid amide) (Khalaj et al. 2018) in autistic youth.

The endocannabinoid system, which helps regulate mood and stress, is altered in autism (Zou et al. 2021), leading to interest in phytocannabinoids (plant‐derived cannabinoids). Cannabidiol (CBD) is a major non‐psychoactive component of cannabis with known anxiolytic and anti‐inflammatory properties (Castillo‐Arellano et al. 2023; Iannotti et al. 2014). Cannabidivarin (CBDV) is a non‐psychoactive structural analog of CBD, also being investigated for its potential to modulate E/I balance (Iannotti et al. 2014). The synthetic cannabinoid Nabilone, a CB1 and CB2 receptor agonist, is being investigated in a Phase 1 trial for severe behavioral problems in adults with intellectual and developmental disabilities, including autism (Lin et al. 2023). While uncontrolled case series and large observational studies using CBD‐rich extracts have reported benefits for disruptive behaviors (Aran and Cayam Rand 2024; Bar‐Lev Schleider et al. 2019), placebo‐controlled trials have yielded mixed results. Evidence for all cannabinoid‐based therapies remains preliminary (Aran and Cayam Rand 2024).

Quality RCTs for serotonergic agents (SSRIs) are lacking (Hollander et al. 2012; King et al. 2009). However, a recent network meta‐analysis (Bahji et al. 2025) found that adjunctive anxiolytic buspirone, a serotonin 5‐HT1A receptor partial agonist, was highly effective for agitation in autistic youth (Ghanizadeh and Ayoobzadehshirazi 2015), supporting the targeting of underlying anxiety.

Finally, non‐invasive neuromodulation techniques like repetitive transcranial magnetic stimulation (rTMS, which uses magnetic pulses to stimulate specific brain regions, distinct from the generalized seizure induction of electroconvulsive therapy, ECT) offer potential to directly modulate the amygdala‐prefrontal circuits (Xiao et al. 2025) implicated in irritability in autistic children (Ibrahim et al. 2019), but evidence remains mixed (Ni et al. 2024; Ni et al. 2023). Similarly, a recent meta‐analysis of transcranial direct current stimulation (tDCS) in autistic youth demonstrated significant behavioral and emotional regulation improvements (Singh et al. 2026), highlighting another promising modality that requires urgent translation to adult trials. For treatment‐resistant, severe, and life‐threatening aggression or SIB, ECT shows strong beneficial evidence, particularly when associated with catatonia or mood disturbances (Wachtel et al. 2025).

## 8 The Critical Role of Non‐Pharmacological and Psychosocial Interventions

A purely pharmacological approach is often insufficient because it fails to address the common functional drivers of irritability—emotion dysregulation, trauma responses, and lagging skill. Lasting change often requires evidence‐based psychosocial interventions that directly teach the skills needed to regulate a sensitized nervous system and address the downstream effects of trauma. However, as established earlier, these approaches may be less effective for behaviors maintained by automatic reinforcement or driven by intrinsic factors (Hagopian et al. 2015). In such cases, interventions targeting the underlying neurobiology may be primary. While a recent systematic review (Nuske et al. 2024) has provided recommendations for strategies like parent‐implemented interventions, emotion regulation training, and antecedent‐based interventions, as with pharmacological research, this evidence base has yet to be extended to adults, leaving clinicians without age‐appropriate guidance.

Applied Behavior Analysis (ABA), particularly through Functional Behavior Assessment, remains the most widely implemented behavioral intervention for identifying and modifying challenging behaviors (Gitimoghaddam et al. 2022; Newcomb and Hagopian 2018). However, its evidence base in adults is extremely limited, and its use is highly contested. Many autistic self‐advocates raise concerns about historical or poorly implemented ABA practices that prioritize compliance and normalization over self‐determination (Leaf et al. 2022), while many caregivers, particularly those supporting individuals with profound autism and dangerous behaviors, view behaviorally informed interventions as essential for managing safety and teaching critical life skills. Furthermore, even when individuals or families desire these behaviorally informed interventions, systemic barriers and a profound lack of adult‐focused providers severely limit access to care. These tensions are driving evolution toward approaches such as Positive Behavior Support (PBS), which utilizes behavioral principles while emphasizing person‐centered, strengths‐based, quality‐of‐life‐focused approaches and environmental modification (Stalford et al. 2024). Importantly, much like parent‐training models, the specific evidence base for PBS in autistic adults remains sparse, relying almost entirely on extrapolation from pediatric cohorts (Durand et al. 2013) and the broader intellectual and developmental disabilities (IDD) literature (McClean et al. 2005). Critically, when addressing irritability, it is essential to recognize that behaviors often targeted for reduction, such as insistence on sameness or repetitive behaviors, may serve an adaptive regulatory function for some autistic individuals, particularly when more cognitively demanding strategies are inaccessible (Cai and Samson 2025; Dell'Osso et al. 2023). Interventions must therefore carefully assess the function of these behaviors before attempting modification.

Beyond environmental modification and skill‐building strategies, interventions delivered directly to autistic adults that target core mechanisms of distress show promise, though the overall evidence base is highly fragmented. Currently, Dialectical Behavior Therapy (DBT), originally developed for borderline personality disorder, possesses the strongest emerging direct empirical support for treating emotion dysregulation in autistic adults without ID (Weiner et al. 2025). Recent RCTs demonstrated that DBT is feasible, acceptable, and effective for autistic adults without ID who experience emotion dysregulation and self‐harming behaviors (Bemmouna et al. 2025). DBT teaches skills in four key areas: mindfulness (enhancing awareness), distress tolerance (surviving crises without making them worse), emotion regulation (understanding and influencing emotions), and interpersonal effectiveness (Linehan and Wilks 2015). The finding that improvements in emotion dysregulation were mediated by a decrease in alexithymia Bemmouna et al. (2025) suggests DBT could be effective because it targets this core deficit in emotional identification. Similarly, mindfulness‐based treatments (MBT), adapted for autistic adults (Spek et al. 2013), may reduce anxiety by increasing emotional awareness and the capacity to cope with strong internal states (Simione et al. 2024).

Conversely, the evidence for interventions targeting other core cognitive drivers of irritability still relies almost entirely on extrapolation from youth. Given the central role of intolerance of uncertainty in driving irritability‐related anxiety (Stark et al. 2021), interventions specifically targeting this construct are crucial. Adapted Cognitive Behavioral Therapy (CBT) approaches (e.g., the CUES program) show promise in youth (Rodgers et al. 2023; Rodgers et al. 2017) and require urgent, direct study in adults.

Whether implementing mechanism‐focused therapies (like DBT or CBT) or PBS frameworks, substantial adaptation is required for autistic adults with ID. Concept simplification alone is insufficient; the entire therapeutic framework must be reimagined. Successful adaptations include replacing abstract emotion concepts with concrete visual representations—using color‐coded “feeling thermometers” or tactile objects to represent internal states (Jones et al. 2021). Skills must be broken into micro‐steps with frequent repetition and real‐world practice. For example, a skill focused on physical well‐being might be adapted into a visual daily checklist with picture symbols. These structured teaching strategies, derived from fundamental principles of behavioral learning science (the foundation of approaches like ABA) (Gitimoghaddam et al. 2022), are essential for skill acquisition in this population. These adaptations require parallel training for direct support professionals to ensure skills are prompted and reinforced consistently.

## 9 The Hidden Crisis: Caregiver Trauma and Burnout

The interventions described above cannot succeed without a parallel focus on those who deliver them. While we reconceptualize part of irritability expression as a stress response in autistic individuals, we must acknowledge the parallel trauma experienced by caregivers navigating these episodes without adequate support. Parents describe living in constant hypervigilance, scanning for triggers while suppressing their own stress responses that might escalate situations (Zablotsky et al. 2013). The physical demands (such as blocking self‐injury, managing property destruction, de‐escalating aggression, and sustaining injuries from assault) take a cumulative toll, with caregivers reporting chronic pain, sleep deprivation, and stress‐related health conditions at rates exceeding combat veterans (Hayes and Watson 2013).

Professional caregivers face additional challenges: inadequate training, minimal supervision, and low wages for managing complex behavioral needs. Staff turnover exceeds 70% annually in many settings, creating instability that exacerbates irritability (Hewitt et al. 2021). The ethical distress of implementing inadequate interventions, watching individuals suffer while lacking tools to help, contributes to compassion fatigue. Any intervention framework must therefore be dyadic, addressing both the autistic individual's regulation needs and the caregiver's capacity to remain regulated while providing support.

To address this dyadic need, caregiver support and training must be a cornerstone intervention. A recent meta‐analysis found parent training for maladaptive behaviors is highly effective for reducing the behavioral manifestations associated with irritability (as measured by scales like the ABC‐I), with an effect size comparable to antipsychotics (Choi et al. 2024). These programs empower caregivers to understand the function of challenging behaviors, modify environmental triggers, and teach their children more adaptive ways to communicate needs and regulate emotions. However, there is a profound lack of research examining the adaptation and efficacy of these models for the caregivers of autistic adults—including aging parents, spouses, and professional support staff. Adapting these evidence‐based models for the adult lifespan, for instance by packaging skills training into accessible formats like video modules and remote coaching for direct‐support professionals (Gerritsen et al. 2019), is an urgent priority.

## 10 An Urgent Call for a New Era of Clinical Trials

The field is at a critical inflection point. We are not limited by a lack of plausible targets, but by a scarcity of high‐quality clinical trials in autistic adults. While meta‐analyses of the existing pediatric evidence base provide valuable synthesis, further progress ultimately requires generating new, high‐quality data in adult populations. To meaningfully advance care, we call for a multi‐pronged strategic shift that differentiates between immediate and long‐term goals. Immediately actionable steps include validating adapted multi‐modal measures (e.g., self‐report plus observational biosensors) in adult cohorts, and prioritizing pilot randomized controlled trials of existing transdiagnostic psychosocial interventions (like DBT). Long‐term goals demand a systemic shift in funding to support large‐scale, biomarker‐driven clinical trials of novel therapeutics designed specifically for autistic adults, moving beyond the metabolic risks of current antipsychotics.

The new generation of trials must be built on four interconnected principles. First, the research agenda must pivot from its focus on children to prioritize well‐designed RCTs in adults (Beasant et al. 2024), including those with profound autism (Thurm et al. 2022), using inclusive methods (Shariq et al. 2023). Second, the research portfolio must diversify beyond antipsychotics to rigorously test promising agents and evaluate the psychosocial interventions outlined above. Third, outcome measures need to be revolutionized, moving beyond the ABC‐I to a multi‐modal battery combining precise affective scales, objective physiological data, and functional outcomes (Beck et al. 2020; Saatchi et al. 2023). Finally, trials should be mechanistically driven and biomarker‐informed, designed to reveal not just whether a treatment works, but how and for whom. To account for the profound idiosyncrasy of the autistic brain, future designs could utilize individual‐level analytical tools (such as dense sampling (McGowan et al. 2023) or N‐of‐1 trials (Augustine et al. 2024)) and non‐Gaussian statistical approaches to capture meaningful biological variance and model person‐specific trajectories (Lin et al. 2026).

The failure to advance these treatments reflects systemic barriers beyond mere research neglect. Pharmaceutical companies face perverse economic incentives: the adult autism market is perceived as small, heterogeneous, and high‐risk, with complex consent issues and high dropout rates deterring investment (Howes et al. 2018). The off‐label use of existing medications provides sufficient profit without the expense of formal trials. Simultaneously, regulatory agencies have not mandated adult studies following pediatric approvals, allowing the extrapolation of pediatric data to continue indefinitely.

Academic researchers face their own hurdles. Funding agencies prioritize early intervention, operating under the untested assumption that treating children will prevent adult difficulties. However, this assumption is contradicted by longitudinal evidence demonstrating the persistence of severe challenging behaviors (Minshawi et al. 2014; Taylor et al. 2011), highlighting the critical need for research focused on lifelong support. The complexities of adult autism research (including heterogeneous presentations and the challenges of recruitment and retention, particularly for those with high support needs) can lead to perceptions of high risk within the grant review process. This creates a self‐fulfilling prophecy where studies are not funded because they are deemed too difficult, thereby perpetuating the evidence gap. The lack of established adult autism research infrastructure, from recruitment networks to specialized outcome measures, makes each study prohibitively expensive and time‐consuming. Furthermore, the severe shortage of general psychiatrists equipped or willing to treat autistic adults exacerbates this “services cliff” (Westover 2024), leaving many stranded without expert pharmacological management. Most perniciously, the field suffers from therapeutic nihilism: the belief that adults are “too late” for meaningful intervention, despite evidence of lifelong neuroplasticity and the profound, ongoing suffering experienced by many adults and their families/caregivers (Howlin and Magiati 2017).

We opened by asking what we are really treating when we prescribe risperidone or aripiprazole for “irritability” in autistic adults. The answer that emerges from the model developed in this review is that, too often, we are treating the behavioral signature of a chronically dysregulated, hyper‐aroused, and adverse‐experience‐laden nervous system—not the underlying psychophysiological state itself. The autistic community has waited long enough. The era of extrapolating from pediatric data and relying on a single class of medication with significant long‐term risks must end. A concerted, collaborative effort between researchers, clinicians, funding agencies, industry, and the autistic community is required to launch a new generation of mechanistically driven, biomarker‐informed, and adult‐focused clinical trials—trials capable of treating the state, not merely silencing its expression—and to thereby improve the lives of autistic adults and their families/caregivers.

## Author Contributions

H.‐Y.L., A.N.W., and M.K.J. contributed to the study conception and design. The first draft of the manuscript was written by H.‐Y.L. and all authors commented on previous versions of the manuscript. All authors read and approved the final manuscript.

## Funding

The authors have nothing to report.

## Conflicts of Interest

All authors have no financial, non‐financial, or competing interests to declare that are relevant to the content of this article. Dr. Lin receives an honorarium for serving as an Associate Editor for *NeuroImage: Clinical*. In the past 36 months, Dr. Jha has received contract research grants from Neurocrine Bioscience, Navitor/Supernus and Janssen Research & Development; honorarium to serve as Section Editor of the Psychiatry & Behavioral Health Learning Network and as Guest Editor for *Psychiatric Clinics of North America* from Elsevier; consultant fees from Janssen Scientific Affairs, Neurocrine, Abbvie, Sanofi, Definium (MindMed), and Boehringer Ingelheim; fees to serve on Data Safety and Monitoring Board for Worldwide Clinical Trials (Eliem, Skye and Inversago), Vicore Pharma and IQVIA (Click); and honoraria for educational presentations from North American Center for Continuing Medical Education, Medscape/WebMD, Clinical Care Options, Soterix Medical Inc., Physicians' Education Resource, Efficient CME, and H.C. Wainwright & Co.

## Data Availability

Data sharing not applicable to this article as no datasets were generated or analysed during the current study.
